# Structure–property relationships of alginate–protein–polysaccharide composites for encapsulation and controlled release of *Lactobacillus bulgaricus* LB12

**DOI:** 10.1016/j.fochx.2026.104149

**Published:** 2026-06-28

**Authors:** Marko Vinceković, Botagoz Mutaliyeva, Sanja Kajić, Nataša Šijaković Vujičić, Anđela Pustak, Suzana Šegota, Tanja Jurkin, Lana Živković-Gemzić, Assem Issayeva, Galiya Madybekova, Usen Akhanov

**Affiliations:** a Division of Agroecology, Department of Chemistry, University of Zagreb Faculty of Agriculture, Svetošimunska 25, 10000 Zagreb, Croatia; b Biotechnology Department, M. Auezov South-Kazakhstan University, Tauke-Khan, Shymkent 160000, Kazakhstan; c Chemistry Department, O. Zhanibekov South-Kazakhstan Pedagogical University, Baitursynov Street, 13, Shymkent 160000, Kazakhstan; d Laboratory for Biocolloids and Surface Chemistry, Ruđer Bošković Institute, Bijenička c. 54, 10000 Zagreb, Croatia; e Division of Organic Chemistry and Biochemistry, Ruđer Bošković Institute, Bijenička c. 54, 10000 Zagreb, Croatia; f Croatian Tissue and Cell Bank, Clinical Department of Transfusion Medicine and Transplantation Biology, University Hospital Centre, Kišpatićeva 12, 10000 Zagreb, Croatia; g Radiation Chemistry and Dosimetry Laboratory, Ruđer Bošković Institute, Bijenička c. 54, 10000 Zagreb, Croatia; h Scientific and Production Enterprise “Antigen” LLP, Village Abai, Karasai District, Almaty Region, Kazakhstan

**Keywords:** Calcium alginate, Composites, *Lactobacillus bulgaricus*, Encapsulation, Green technologies, Agriculture

## Abstract

This study investigates alginate-based composites for encapsulating the probiotic strain *Lactobacillus delbrueckii* ssp. *bulgaricus* (LB12). Alginate/casein, alginate/carrageenan and alginate/casein/κ-carrageenan composites and their formulations with LB12 were prepared by ionic gelation using calcium chloride. Combined analyses (FTIR, rheological, thermal, morphological, and bacterial cell release from formulations) reveal that the microparticle network structure and physicochemical properties depend on complex interactions among constituents. FTIR spectra confirm the formation of crosslinked composites via hydrogen bonds and electrostatic interactions. Bacterial encapsulation weakened hydrogen bonds and electrostatic interactions between the composite components, thereby increasing the degree of swelling. All microparticles exhibit good mechanical and thermal stability consistent with their well-organized microstructures. Variations in storage modulus and yield strength among microparticles are consistent with morphological differences observed by microscopy, where increased formulation complexity and bacterial incorporation result in more heterogeneous, less compact particle surfaces. In vitro release studies demonstrated an initial lag phase followed by diffusion-controlled bacterial cells release from alginate/casein/LB12 formulation and swelling/relaxation of the polymer matrix for the alginate/casein/carrageenan/LB12 formulation. The findings highlight the synergistic role of alginate, casein, and carrageenan in developing effective carriers for the delivery of bacterial cells in functional food, nutraceutical and agricultural applications.

## Highlights

• Alginate composites formed via hydrogen bonding and electrostatics.
• Casein and carrageenan modulate network structure and swelling.
• Encapsulation alters intermolecular interactions and gel mechanics.
• Composite complexity reduces modulus but enhances swelling capacity.
• Distinct release mechanisms: diffusion vs matrix relaxation.

## Graphical abstract

**Figure:**
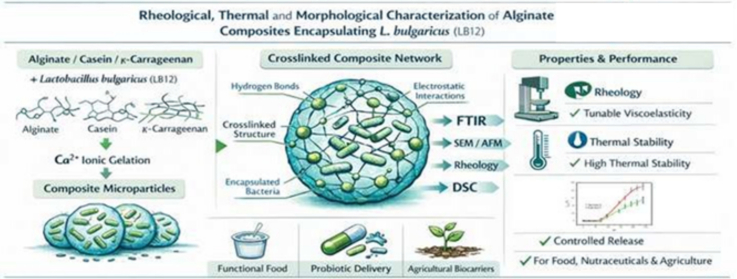

## 1 Introduction

Lactic acid bacteria (LAB) are versatile microorganisms found in various natural sources and have a wide range of applications in healthcare, food production, and biotechnology. They contribute to human health as probiotics, prebiotics, in disease prevention, and mental health. LAB are used in a variety of applications, from the food industry (fermentation, preservation, contribution to flavor and texture), pharmaceutical and cosmetic industries, biotechnology (biopreservation, bioconversion), to agriculture (plant health, animal feed) (Murata et al., 2025). Due to their multiple beneficial effects on soil and plant health, LAB are gaining importance as biofertilizers and biocontrol agents to promote plant growth (Raman et al., 2022). As biofertilizer, LAB can promote biodegradation, improve nutrient solubilization, and increase soil fertility, and as biocontrol agents suppress the growth of plant pathogens. Additionally, LAB are among the most commonly used bacteria as probiotics in aquaculture (Mariom et al., 2025). The main limitation of LAB in application is their ability to survive in the required environments (Ibáñez et al., 2023).

A well-known species of lactic acid bacteria, *Lactobacillus bulgaricus*, is essential for the production of fermented dairy products, especially yogurt. Along with *Streptococcus thermophilus*, this strain is key to the fermentation process that gives yogurt its distinctive taste, texture and health benefits. Probiotics, such as *Lactobacillus bulgaricus*, are known for numerous health benefits and for their ability to enhance food preservation. They can strengthen the immune system, improve gut health, and provide treatment benefits for a range of gastrointestinal conditions (Liu et al., 2016). Despite its significance, maintaining the viability of *L. bulgaricus* during processing, storage and application remains a considerable challenge. The *Lactobacillus bulgaricus* cells are sensitive to oxygen, acidity, bile salts, temperature and osmotic stresses, which can compromise their survival (Amund, 2016). To address these issues, microencapsulation approaches based on protective polymer matrices have emerged as effective strategies. The main advantages of encapsulating lactic acid bacteria in food production are enhanced bacterial survival, extended shelf life, and improved delivery of probiotic benefits. This technique is particularly valuable in dairy products, fermented foods, and functional beverages. The encapsulated probiotic can be incorporated into a wide range of foods without affecting flavor, texture, or appearance.

Alginate hydrogel microparticles, which provide a favorable environment for probiotic protection, are widely recognized as useful polysaccharides for probiotic delivery systems. However, despite the significant advantages of alginate gel for encapsulation, it also has disadvantages, including reduced acid resistance, relatively low mechanical properties, and surface porosity. Recent developments in the development of composite carriers, particularly those combining alginate with polysaccharides and proteins, have shown encouraging results in enhancing the viability and encapsulation efficiency of Lactobacillus (Ramdhan et al., 2020; Wang et al., 2022). These composite systems ensure the strain's probiotic functions by enhancing its resilience in challenging environments, while also providing structural integrity (Vinceković et al., 2024). Casein is food-safe and widely used as a carrier for encapsulating Lactobacillus bacteria, thereby improving their survival during storage and passage through the gastrointestinal tract (Vinceković et al., 2024). It is often combined with other biopolymers to form microcapsules or edible films that protect probiotic bacteria from harsh conditions. An effective material for encapsulating probiotic bacteria is an alginate/casein composite that offers improved environmental protection, enhanced viability, and controlled release (maintaining stability in acidic environments and releasing in neutral environments) (Li et al., 2021; Ramdhan et al., 2020; Sadiq et al., 2021). κ-carrageenan, a sulfated polysaccharide from red seaweed, is also employed in probiotic delivery systems. It is widely used as a protective encapsulation material and stabilizer in dairy systems to improve the survival, delivery, and functionality of *Lactobacillus bulgaricus* (Büyükgüngör, 2007). However, its gels are often mechanically weaker, prompting their use in combination with a stiffer matrix, such as alginate (Koh et al., 2022). Alginate and κ-carrageenan interact primarily through hydrogen bonding and electrostatic forces, forming composite gels with enhanced mechanical, thermal, and functional properties (Ye et al., 2017). Alginate/carrageenan composites have been shown to improve probiotic viability in dairy matrices, with encapsulation efficiencies of up to 95%. (Saeed et al., 2024). Briefly, alginate/casein or alginate/carrageenan composites provide multifunctional protection, ensuring that probiotics remain viable until they reach the gut, where they can deliver their health benefits (Dong, Chen, Xin, Qin, Cheng, Shi, & Tang, 2013; Sıçramaz et al., 2025). Integrating alginate, casein, and carrageenan into a composite system leverages their complementary physicochemical and functional properties: alginate provides structural integrity, casein provides protein-mediated stability, and carrageenan provides controlled release. The inclusion of casein in such composites remains underexplored but may be synergistic. This study investigates the physicochemical properties of alginate-based carriers (with casein and casein/κ-carrageenan) for encapsulating *Lactobacillus bulgaricus*, in light of the demand for innovative matrices with tailored release kinetics. Our research aims to clarify how the addition of casein and carrageenan affects probiotic delivery efficiency by systematically characterizing the carrier's fundamental properties. This will ultimately contribute to the development of sustainable green biofertilizer systems for hydroponic and functional food production by improving the stability, viability, and controlled delivery of beneficial probiotic LB12 microorganisms.

## 2 Materials and methods

### 2.1 Materials

Alginic acid sodium salt (SA) (CAS Number: 9005-38-3, molecular weight 280,000 in g/mol, M/G ratio of approximately 1.56), kappa carrageenan (CAR) (CAS Number: 11114–20-8, molecular weight 788.65 g/mol) and Calcofluor white M2R (CAS Number.:4193-55-9, molecular weight 960.95 in g/mol) were provided by Sigma Aldrich (St. Louis, MO, USA). Grammol (Zagreb, Croatia) supplied sodium caseinate (CA) (CAS Number: 9005-46-3), while Kemika (Zagreb, Croatia) supplied the commercial CaCl_2_ (CAS Number: 10043-52-4). The remaining chemicals were all analytical grade and used exactly as supplied; none needed further purification.

#### 2.1.1 *Lactobacillus delbrueckii* ssp. *Bulgaricus* cells and preparation for encapsulation

Chr. Hansen (HĿnsholm, Denmark) provided the *Lactobacillus delbrueckii* ssp. *bulgaricus* (LB12) culture, which was freeze-dried, activated, and homogenized for two hours at 37 °C. Furthermore, during encapsulation, 1 g/100 mL of LB12 cells yielded functional biomass (9–9.5 log CFU/mL).

#### 2.1.2 Microparticles preparation

Microparticles were prepared in a single step via ionic gelation at room temperature under sterile conditions. Several sets of microparticles were prepared: calcium alginate as control microparticles (Sample 0), composite microparticles: calcium alginate/casein (Sample 1), calcium alginate/κ-carrageenan (Sample 2) and calcium alginate/casein/κ-carrageenan (Sample 3), and microparticle formulations loaded with LB12 calcium alginate/LB12 (Sample 4), calcium alginate/casein/LB12 (Sample 5), calcium alginate/κ-carrageenan/LB12 (Sample 6) and calcium alginate/casein/κ-carrageenan/LB12 (Sample 7). Sample 0 was prepared by dripping 100 ml of SA solution (2% w/v) into 100 mL of calcium chloride solution (2% w/v). Composite microparticles were prepared by dripping a mixture of SA(2% w/v)/CA(1% w/v), SA/(2% w/v)/CAR(1% w/v) or SA(2% w/v)/CA(1% w/v)/CAR(1% w/v) into 100 mL of calcium chloride solution (2% w/v).

Microparticle formulations were prepared as follows: freeze-dried LB12 cells (1 g) were immediately added to 100 mL of SA(2% w/v), SA(2% w/v)/CA(1% w/v), SA(2% w/v)/CAR(1% w/v) or SA(2% w/v)/CA (1% w/v)/CAR(1 w/v%) mixtures and homogenized by slight mixing for 10 min (Biosan Orbital Shaker-Incubator ES-20, Medical-Biological Research & Technologies, Riga, Latvia). The mixture was added to 100 mL of a 2% (w/v) CaCl_2_ solution. The experiment used a 450 μm encapsulator nozzle operated at 600 Hz and 121 mbar (Encapsulator Büchi-B390, Büchi Labortechnik AG, Flawil, Sankt Gallen, Switzerland) with steady magnetic stirring. To promote gel strengthening, the generated microparticles were kept at room temperature for an additional 30 min. To remove excess CaCl_2_, microparticles were rinsed three times with sterile distilled water, filtered through a Büchner funnel, and stored at 4 °C for future studies.

### 2.2 Methods

#### 2.2.1 Size and charge determination of LB12 cells suspended in water and calcium ion solutions

Dynamic light scattering (DLS) measurements were performed using a Zetasizer Ultra (Mal*v*ern Panalytical, London, UK) equipped with a 632.8 nm He—Ne laser to ascertain the hydrodynamic diameter of nanoparticles suspended in solutions of calcium ions and water (concentrations ranging from 1 to 4% w/v). The Multi-Angle Dynamic Light Scattering (MADLS®, Netanya, Israel) technology was used to assess the samples at three scattering angles (front: 13°, side: 90°, back: 173°). The data were then compiled into a single integrated measurement. DTS0012 1 cm plastic cuvettes were used for the measurements. The averages of three to five measurements were used to determine hydrodynamic diameters using the number distributions. The same apparatus was used to detect zeta potentials via electrophoretic light scattering in DTS1070 folded capillary cells. The mean of three measurements is used to represent the zeta potential.

#### 2.2.2 Microscopic observations

Several microscopic techniques were used to examine LB12 (colonies and cells) and microparticles: (i) optical microscope (OM) (Leica MZ16a stereomicroscope, Leica Microsystems Ltd., Balgach, Switzerland), (ii) scanning electron microscope (SEM) (FE-SEM, model JSM-7000 F, Jeol Ltd., Akishima, Japan), and (iii) and atomic force microscope (AFM) (MultiMode Scanning Probe Microscope with Nanoscope IIIa Controller (Bruker Billerica, USA). The average diameter of wet and dry microparticles was measured with optical microscopy using Olympus Soft Imaging Solutions GmbH, version E_LCmicro_09Oct2009. To determine the size distribution, 50 microparticles were randomly selected from triplicate batches. The LB12 colony size and cell shape were assessed using Olympus Soft Imaging Solutions GmbH (version E_LCmicro_09Oct2009) and optical microscopy.

Dried samples for SEM analysis were placed on highly conductive graphite tape. The FE-SEM was attached to an EDS/INCA 350 (energy dispersive X-ray analyzer) designed by Oxford Instruments Ltd. (Oxon, UK). ImageJ (Phyris, version 9.1.0.0198) was used to measure pore size on a microparticle surface.

All AFM samples were imaged in tapping mode under ambient conditions. R-TESPA 300 probe (Bruker, Billerica, USA, Nom. Freq. 300 kHz, Nom. spring constant of 40 N/m) was used for imaging. The tapping force was maintained at a low force (0.8–0.9) suitable for the study of soft samples. Imaging and data acquisition were performed with 256 pixels (256 × 256). Images are analyzed using offline AFM NanoScopeTM software (Digital Instruments, Santa Barbara, CA, Version V614r1 and V531r1). All images are shown as raw data, with the exception of the two-dimensional first-order flattening.

#### 2.2.3 Attenuated total reflectance Fourier transform infrared spectroscopy

Each sample was evaluated using the attenuated total reflectance (ATR-FTIR) recording technique in combination with Fourier transform infrared spectroscopy. The materials' FTIR-ATR spectra were obtained using a Cary 660 FTIR spectrometer (Agilent Technologies, Palo Alto, CA, USA) and the Golden Gate single-reflection diamond ATR accessory (Specac). The spectra were recorded in transmission mode in the mid-infrared region (4000–500 cm^−1^).

#### 2.2.4 Swelling degree, loading capacity, encapsulation efficiency, and in vitro LB12 release from microparticles

Detailed protocols for determining encapsulation efficiency (EE), loading capacity (LC), swelling degree (S_w_), and the fraction of released LB12 (f_LB12_) from microparticle formulations have previously been reported (Vinceković et al., 2016).

(a) Encapsulation efficiency

Encapsulation efficiency is the ratio of encapsulated substance detected in microparticle formulations to the initial concentration used to form the microparticle. The following formula was used to compute encapsulation efficiency, defined as the percentage of live cells used in the encapsulation process.

(1) $$\mathrm{EE}\;\left(\%\right)=\left(N_{\mathrm{com}}/N_{\mathrm{sus}}\right)\times 100$$

where N_com_ (number of colony-forming units (CFU) per gram of microparticles) and N_sus_ (number of CFU per gram of solution) are viable counts in composites and cell suspension utilized for encapsulation, respectively. The original cell culture used for encapsulation was serially diluted (1,10) in sterile saline solution (0.85% NaCl, Merck, Germany), duplicated on MRS agar plates (Biolife, Milan, Italy), and incubated at 30 °C for 48 h under anaerobic conditions.

(b) Loading capacity (LC)

The loading capacity of a microparticle formulation refers to the amount of encapsulated material loaded per unit weight. The loading capacity was evaluated by dissolving 1 g of dry microparticle formulations in 10 mL of a combination of 0.2 mol/dm^−3^ NaHCO_3_ and 0.06 mol/dm^−3^ Na_3_C_6_H_5_O_7_ × 2H_2_O (Vinceković et al., 2016). The resulting solution was filtered, and the cell number was determined spectrophotometrically (Shimadzu 1900, Kyoto, Japan) at λ = 600 nm using the method of Waghunde et al. (Waghunde et al., 2010). The loading capacity was expressed as the number of *LB12* cells per 1 g of dry microparticles (CFU g^−1^) and calculated by the following equation:

(2) $$\mathrm{LC}=c\left(\mathrm{LB}12\right)\times \left(V/w\right),$$

where $c\left(\mathrm{LB}12\right)$ is the number of LB12 cells per 1 g, Vis the volume of citric buffer in liters, and wis the weight of microparticles.

(c) Swelling degree

The degree of swelling indicates the extent to which alginate hydrogels absorb and swell in an aqueous environment. The swelling degree was computed using the following equation:

(3) $$S_{w}=\left(\frac{w_{t}-w_{0}}{w_{0}}\right)\times 100$$

where w_t_ is the weight of the swollen microparticles and w_0_ is their initial weight.

(d) In vitro release of LB12 from microparticle formulations

The release of LB12 cells from microparticle formulations was investigated at ambient temperature. Microparticle formulations (10 g) containing LB12 cells were dispersed in 100 mL of deionized water and left to stand undisturbed throughout the trials. At regular intervals, the dispersion was stirred for 60 s, aliquots were collected, and cell numbers were measured spectrophotometrically (Shimadzu 1900, Kyoto, Japan) at λ = 600 nm. The results are provided as the fraction of LB12 cells discharged using the equation below:

(4) $$f_{\mathrm{LB}12}=R_{t}/R_{\mathrm{tot}}$$

where f_LB12–12_ represents the fraction of LB12 cells released, R_t_ is the amount of LB12 cells released at time t, and R_tot_ is the total amount of LB12 cells in the microparticle formulations.

Experiments were conducted in triplicate at room temperature. IBM SPSS Statistics 22 and Microsoft Excel 2016, with the XLSTAT statistical add-in, were used to analyze the collected data. The mean value and standard deviation were displayed for every data point.

#### 2.2.5 Rheological measurements

The rheological characteristics of the microcapsules were analyzed through oscillatory rheology. The storage (G′) and loss (G″) moduli were determined using a mechanical rheometer (Anton Paar MCR 302, Stuttgart, Germany) with a sandblasted steel plate−plate geometry (PP25/S, Anton Paar, Graz, Austria) and a gap of 1.5 mm. The instrument was equipped with a true-gap system, and data were collected using RheoCompass software. Adhesive tape was applied to the base plate to prevent the microcapsules from slipping during the measurements. Sample temperature was regulated using a Peltier temperature control located at the base of the geometry, along with a Peltier-controlled hood (H-PTD 200). During the experiment, a sample was placed on the rheometer's base plate, and the plate was adjusted using the software's true-gap function. After allowing 2 min at 23 °C, measurements of G′ and G″ moduli were consistently taken within the linear viscoelastic region (LVR). The yield stress was determined by conducting a strain (γ) sweep between 0.01% and 100% at a constant frequency of 10 rad/s. The rheological properties of the sample were found to be strain-independent up to the yield strain. Beyond the yield strain, the rheological behavior became nonlinear. Subsequent frequency sweeps (0.1–100 rad/s) were performed at 23 °C, maintaining a strain of 0.1% within the LVR to explore the time-dependent deformation behavior of the sample. Amplitude sweep tests were performed at room temperature. All rheological measurements were performed in triplicate. Storage modulus (G′), yield stress and yield strain values are reported as mean ± standard deviation (SD), calculated from three independent measurements. The variability of the loss factor was negligible, with a coefficient of variation (CV) of approximately 2% and a typical standard deviation in the range of ±0.002–0.004. Therefore, SD values for the loss factor are not presented in the tables.

Representative amplitude sweep curves for each sample are shown in the figures, whereas the rheological parameters summarized in the tables are reported as mean ± SD values obtained from replicate measurements.

#### 2.2.6 Differential scanning calorimetry (DSC)

Thermal properties of the studied samples were examined with differential scanning calorimetry DSC on a Perkin Elmer Diamond Calorimeter calibrated with indium and zinc standards in dynamic mode. All microparticles, with and without LB, were dried, and samples weighing between 3.06 and 5.55 mg were sealed into aluminum pans. For each sample, heating and cooling cycles were measured from 25 °C to 350 °C under a pure nitrogen atmosphere at 10 °C/min. The peak maxima and corresponding enthalpies, as well as the possible glass transition temperatures, were calculated from heating cycles using Pyris Software (Version 11) for a Perkin Elmer DSC and were normalized prior to analysis.

## 3 Results and discussion

The results are presented and discussed in two sections. The first part analyzes the morphology of colonies and LB12 cells and their interactions with calcium ions. In the second part, the physicochemical properties of the prepared microparticles, which are essential for the controlled release of bacteria, were evaluated.

### 3.1 Morphology of *Lactobacillus delbrueckii* ssp. bulgaricus and interactions with calcium ions

#### 3.1.1 Morphology of *Lactobacillus delbrueckii ssp. bulgaricus* colonies and cells

*Lactobacillus delbrueckii ssp. bulgaricus* is classified as Gram-positive because its cell wall contains a strong peptidoglycan layer that maintains a crystal violet colour throughout the Gram staining method (Teixeira, 2014). Calcium ions are among the essential metal ions that support the growth of Lactobacillus and increase their resistance (Mrvčić et al., 2012). It was shown that encapsulating Lactobacillus bacteria in the same compartment as calcium does not inhibit their activity and, in fact, creates favorable conditions for their propagation (Jurić et al., 2020; Vinceković et al., 2025).

**Fig. 1:**
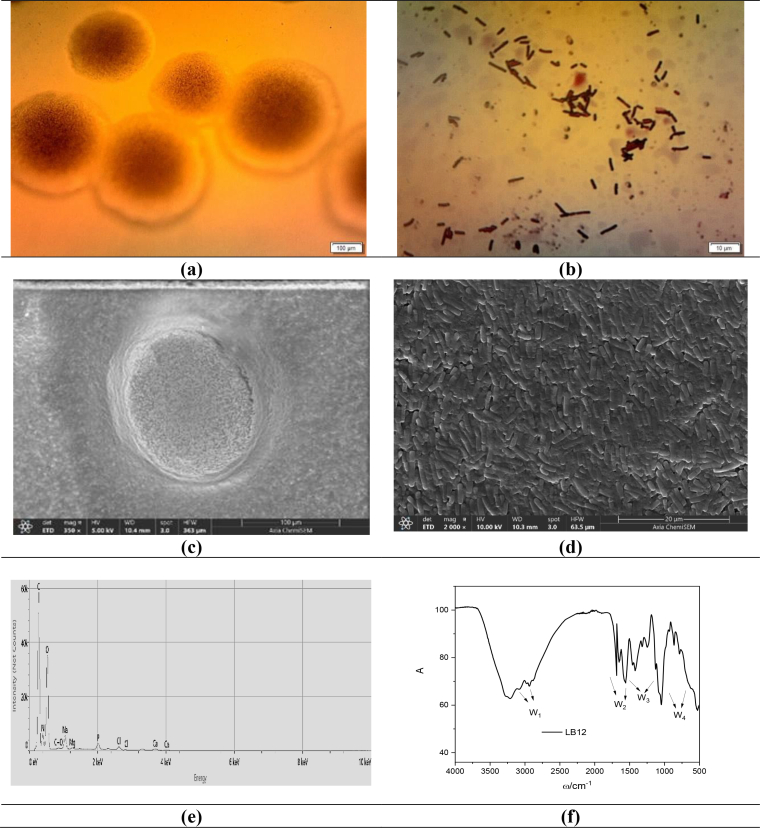
OM microphotographs of LB12 (a) colonies and (b) cells, and (c) SEM microphotographs of lyophilized LB12 (c) colonies and (d) cells. The scale bars are indicated on each image. (e) EDS semi-quantitative analysis of LB12 colonies (expressed in atomic weight percent) and (f) FTIR spectra of LB12.

Fig. 1 shows OM and SEM micrographs of LB12 colonies and cells, as well as EDS and FTIR analyses. LB12 colonies appeared as small, off-white to cream-colored with a slightly irregular or rough surface texture (Figs. 1a,c) (Tumbarski et al., 2021). The average colony size was around 500 ± 100 μm. Microphotographs of LB12 cells showed a characteristic rod-shaped form (Figs. 1b, d). The average length of the LB12 cells was 6.3 ± 1.3 μm (Teixeira, 2014). The cells seem to be arranged in clusters or short chains. The overall texture of the colony appears dense and somewhat granular (Holzapfel & Wood, 2012). A semi-quantitative EDS analysis of the surface of LB12 colonies revealed the highest percentages of carbon, oxygen, nitrogen, and phosphorus (Fig. 1e). These elements indicate the presence of essential biomolecules such as proteins, nucleic acids, and carbohydrates, providing insight into the structure of LB12 colonies.

FTIR spectrum of LB12 presented in Fig. 1f shows the characteristic wavelength regions of lactic acid bacteria (Dziuba et al., 2007). There is a band at around 3250 cm^−1^ attributed to OH-stretching vibrations mainly arising from the excipient sucrose. In the fatty acid region (marked as W_1_), there are small peaks at 2938 and 2884 cm^−1^, which could denote the asymmetric and symmetric lipid CH_2_ stretching vibrations, respectively (Kummerle et al., 1998). Also, there are peaks characteristics for the amide region (marked as W2) at 1690 cm ^1^ attributed to C=O stretching vibrations of esterified lipids and fatty acids, and two peaks at 1651 cm^−1^ and 1560 cm^−1^ attributable to amide I and amide-II bands arising from endogenous proteins (Kansiz et al., 1999). The spectra from 1500 to 1200 cm-1, marked as W3, are attributed to the mixed region (proteins, fatty acids, and phosphate-bearing compounds). The region between 1321 and 1126 cm^−1^ can be assigned to C–O–C, C—O stretch (carbohydrates, polysaccharides) and at 1090 cm^−1^ to PO₂^−^ stretch (nucleic acids). The region labeled W4 is the polysaccharide region (absorption bands of carbohydrates present within the cell wall resembling a fingerprint), and from 900 to 700 cm-1 - the fingerprint region containing bands that cannot be attributed to specific functional groups (Dziuba et al., 2007), which can be used as a ‘fingerprint’ of the sample (Wolkers & Oldenhof, 2005).

**Fig. 2:**
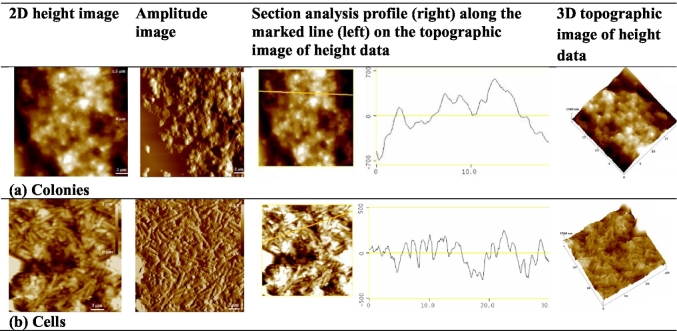
Surface morphology of LB12 colony suspensions and (b) LB12 cells obtained by AFM: (a) 2D height image; (b) amplitude image; (c) section analysis profile (right) along marked line (left) on the topographic image of height data and (d) 3D topographic image of height data on the (20 × 20) μm^2^ surface area.

The surface morphology of LB12 colonies, as observed by AFM (Fig. 2a), reveals a densely packed accumulation of cells, some of which are vertically stacked to a height of 1.3 μm. The cells in the colony are densely packed, so that no individual rod-shaped cells are visible on the colony's surface; only their clusters are visible. Within the colony itself, the cells are densely but homogeneously packed, and the cell clusters are grouped in all three directions on the mica surface, similar to the individual cells in Fig. 2b, where the morphologies of the individual LB12 cells can be seen. AFM micrographs of LB12 cells are shown in Fig. 2b. The size and morphology of LB12 cells determined by SEM (Fig. 1d) were further confirmed by AFM. The rod-shaped cells, with a length of l = (4–6) μm and a diameter of d = (770 ± 50) nm, are homogeneously distributed on the mica surface, with the rods oriented in all three directions. Cell morphology does not indicate any damage or deformation.

#### 3.1.2 Effect of calcium ion concentration on the growth of LB12 colonies on agar plates in the nutrient medium

**Fig. 3:**
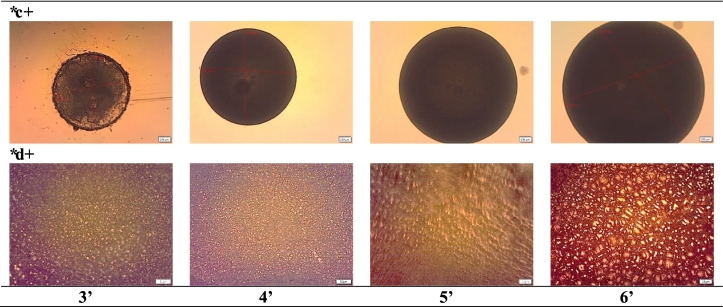
OM micrographs of changes in (a) size and (b) morphology of LB12 colonies, with increasing CaCl_2_ concentration from 1% to 4% (w/v). Scale bars are indicated.

Calcium ions play a structural and regulatory role in *Lactobacillus bulgaricus*. They influence cell morphology by promoting the transition from long chains to short rods and can also modulate the activity of lactic acid bacteria during antifungal interactions (Huynh et al., 2025). Understanding calcium's role in L. *bulgaricus* is important for yogurt production, probiotic efficacy, and industrial fermentation. Fig. 3 presents the effect of calcium ion concentration in the nutrient medium on the size and morphology of LB12 colonies. The increase in the size of colonies from 1543 ± 95 μm (1% CaCl_2_) to 2377 ± 91 μm (4% CaCl_2_) confirms the stimulation of *Lactobacillus* species growth by calcium ions. It is assumed that the studied species, *Lactobacillus bulgaricus*, has Ca^2+^ binding sites that facilitate cell interactions involved in biofilm and colony formation (Huynh et al., 2025). Changes in the surface morphology of the colonies shown in Fig. 2b reveal a denser, more compact cell arrangement as the CaCl_2_ concentration increases. This could be explained by the interaction of calcium ions with the negatively charged components of bacterial cell walls (e.g., lipoteichoic acids), leading to increased cell-to-cell adhesion and aggregation by forming ionic bridges (King et al., 2020).

#### 3.1.3 Effect of calcium ion concentration on LB12 cells in suspensions

**Fig. 4:**
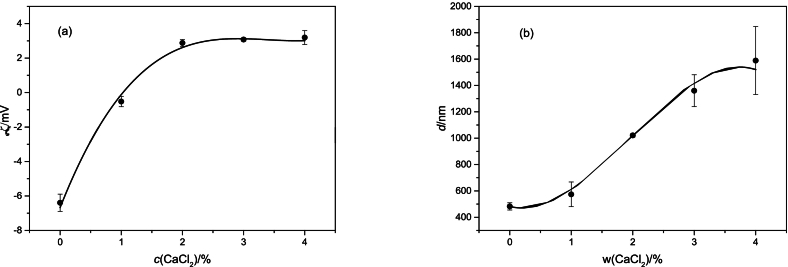
Change in (a) zeta potential (ζ) and (b) size (*d*) of LB12 cell aggregates with increasing CaCl_2_ concentration from 1% to 4% (w/v). Error bars indicate the standard deviation of the means. Insert: corresponding OM microphotographs.

The difference in electrical potential between the surface of a microorganism and the surrounding medium is known as the ζ potential. It measures the net distribution of electrical charge on the surface of bacteria, and several authors have documented its sensitivity in identifying damage and metabolic alterations (Tymczyszyn et al., 2008). The influence of increasing calcium concentration on the zeta potential of LB12 cells is shown in Fig. 4a. The zeta potential of LB12 cells suspended in sterile distilled water was about −6.5 ± 3.3 mV, which is consistent with other studies (Zupančič et al., 2019). Lactic acid bacteria's cell wall is mostly composed of proteins, polysaccharides, peptidoglycans, and (lipo)teichoic acids (Schär-Zammaretti & Ubbink, 2003). Metal ions are bound to cell walls and extracellular polysaccharides by both living and dead cells. This process is influenced by the type and concentration of metal ions, surface charge, metal cations, and ligands, and the accessible functional groups on the cell surface (Vinceković et al., 2024). Main functional groups of LB12 (hydroxyl, carboxyl, amino, and phosphate groups) are associated with its cell wall, surface proteins, and metabolic enzymes (Teixeira, 2014). The addition of calcium chloride to the suspension reduced the negative zeta potential of *Lactobacillus bulgaricus* cells due to calcium binding. In parallel with the decrease in negative zeta potential values, the overall particle size increased due to the reduction in cell repulsion and their aggregation (Fig. 4b). The results clearly show that increasing concentrations of Ca^2+^ significantly enhance the aggregation of LB12 cells. This behavior highlights the critical role of calcium ions in mediating cell adhesion, with implications for a range of biological and industrial applications (Kolodkin-Gal et al., 2023).

### 3.2 Physicochemical characterization of microparticles

#### 3.2.1 Molecular interactions between microparticle constituents

The spectra of both polysaccharides, sodium alginate and κ-carrageenan, and protein, casein, are very well described in the literature. Sodium alginate is an anionic polyelectrolyte built from consecutive groups of two repeating monosaccharide units (mannuronic and guluronic acids) that exhibit characteristic stretching vibrations of carboxylate groups (COO^−^) at 1595 and 1412 cm^−1^. Additional main features are several peaks around 3345 cm^−1^, corresponding to stretching vibrations of the −OH groups, and C—H stretching vibrations at 2932 cm^−1^. The spectral region from 900 to 1200 cm^−1^ indicates the polysaccharide structure, and the strong peak at 1024 cm^−1^ corresponds to the stretching vibration of C-O-C groups (Vinceković & Vlahoviček-Kahlina, 2023). According to the literature, the prominent casein bands are the region around 1649–1650 cm^−1^ assigned to C=O stretching of peptide bonds (amide I), the region around 1510–1580 cm^−1^ for N—H bending and C—N stretching (amide II band), and the region around 1240–1300 cm_−1_ arising from N—H bending and C—N stretching vibrations of the peptide backbone (amide III band) (Singh et al., 2014). As was previously shown in our work casein displays distinctive absorption peaks at the following wavenumbers: at 3283 cm^−1^ and 3069 cm^−1^ (overlapped peaks of O—H and N—H stretching vibrations), at 2967 cm^−1^, 2924 cm^−1^ and 2874 cm^−1^ (C—H stretching vibration peaks), at 1627 cm^−1^ (C=O stretching of peptide bonds, amide I band), at 1516 cm^−1^ (N—H bending and C—N stretching, amide II band) and from 1441 to 1238 cm^−1^ of the peptide backbone (amide III bands) (Kolar et al., 2022). κ-carrageenan is a sulfated polyelectrolyte consisting of repeating disaccharide units (b-(1–3)-D-galactose-4-sulfate and (1–4)-3,6-anhydro-D-galactose) with a strong and broad absorption band around 3250 cm^−1^ attributed to stretching vibrations of -OH groups and at 2924 cm^−1^ to stretching vibrations of C—H groups. The characteristics of the CAR spectrum are very strong absorption bands in the region 1210–1260 cm^−1^ attributed to asymmetric stretching of ester sulfate groups (O=S=O), and in the region 1027–1080 cm^−1^ to glycosidic bonds (C-O-C). The band between 840 and 850 cm^−1^ is the result of sulfation at C-4 of galactose, and the signals recorded at 808 and 790 cm^−1^ is the result of 3,6-anhydrogalactose-2-sulfate (Vinceković et al., 2008).

**Fig. 5:**
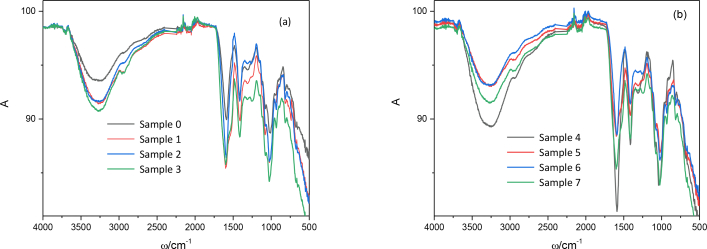
FTIR spectrum of (a) microparticles: calcium alginate (Sample 0), calcium alginate/casein (Sample 1), calcium alginate/κ-carrageenan (Sample 2), calcium alginate/casein/κ-carrageenan (Sample 3), and (b) microparticle formulations: calcium alginate/LB12 (Sample 4), calcium alginate/casein/LB12 (Sample 5), calcium alginate/κ-carrageenan/LB12 Sample 6) and calcium alginate/casein/κ-carrageenan/LB12 (Sample 7).

FTIR spectra of microparticles, calcium alginate and its composites, are presented in Fig. 5a. The strong and broad absorption band between 3500 and 3000 cm^−1^ recorded in the spectrum of calcium alginate (Sample 0) belongs to stretching vibrations of the hydroxyl groups. Besides bending vibrations for hydroxyl groups, several characteristic alginate peaks are at 1567 and 1406 cm^−1^ (asymmetric and symmetric peaks of COO^−^ groups) and at 1074 cm^−1^ for asymmetric −COC and at 1013 cm^−1^ for symmetric −CH_3_ deformation (Jurić et al., 2019).

The spectrum of Sample 1 shows a superposition of alginate and casein characteristic peaks, with visible shifts and intensity changes, as well as the disappearance of individual casein peaks due to interactions between the functional groups of alginate and casein. The absorption band around 3300 cm^−1^, attributed to −OH groups and − NH stretching, becomes broader and more intense, indicating enhanced hydrogen bonding. Peaks at 2967 and 2093 cm^−1^ correspond to C—H stretching of alkane groups, mainly from sodium alginate. Peaks at 1601 and 1422 cm^−1^ reflect both protein and polysaccharide contributions, indicating hydrogen bonding between casein's amide groups and alginate's carboxyl groups. The characteristic alginate peak associated with the symmetric stretching of COO^−^ groups is more intense and somewhat shifted, which reflects electrostatic repulsions between negatively charged casein regions due to phosphate groups on serine residues (pronounced above its isoelectric point (Liu & Guo, 2008)) and free parts of alginate chains (the zeta potential of the calcium alginate matrix is about −10 *V* (Rokstad et al., 2014)). Strong C—O stretching at 1026 and 934 cm^−1^ is characteristic of alginate. The superposition of alginate and casein peaks in the spectrum of Sample 1, with broadened or merged peaks, indicates strong intermolecular interactions, suggesting the formation of a composite network (Vinceković et al., 2024). The spectrum of Sample 2 shows characteristic peaks of both polysaccharides (alginate and κ-carrageenan). Compared to the spectra of Sample 0, the absorption band around 3300 cm^−1^ is broader and more intense. Broadening of the O—H stretching suggests hydrogen bonding among hydroxyl, carboxyl, and sulfate groups. Peaks of the asymmetric and symmetric of carboxylate (COO^−^) groups are somewhat shifted and more intense, indicating electrostatic repulsions between two anionic polysaccharides. Peak shifts and changes in intensity in the carboxylate/sulfate region are caused by the overlap of the sulfate ester peaks from carrageenan with the carboxylate from alginate. The coexistence of carboxylate and sulfate peaks in FTIR spectra, along with peak shifts and broadening, indicates the formation of a composite. The FTIR spectra of Sample 3 show characteristic peaks of polysaccharides (alginate, carrageenan) and proteins (casein). Compared to other composites, the absorption band around 3277 cm^−1^ is the broadest and most intense, indicating enhanced hydrogen bonding. The shifting of the ester sulfate group of carrageenan from 1274 to 1257 cm^−1^ indicates the interaction between casein and carrageenan. The interactions are mainly electrostatic between the –NH_3_^+^ of CA and the –OSO_3_^−^ of CAR (Tang et al., 2021). The broadening and increasing intensity of the O—H stretch, the shifting of the alginate COO^−^ stretch with casein negatively charged side groups of amino acids, the broadening and shifting of the amide bands (I, II, II) with polysaccharides, and the overlapping of the sulfate ester peaks from carrageenan (S=O, C–O–S) with alginate COO^−^ confirm the formation of a crosslinked composite by hydrogen bonds and electrostatic interactions.

FTIR spectra of microparticle formulations are presented in Fig. 5b. The absence of LB12 characteristic bands in the spectra of microparticle formulations confirms successful encapsulation*. Lactobacillus delbrueckii* subsp. *bulgaricus* has a cell surface composed of peptidoglycan, teichoic acids, and proteins, which can carry positive or negative charges depending on environmental conditions. The spectrum of Sample 4 shows that all characteristic alginate peaks are broadened, more intense, and somewhat shifted, indicating enhanced electrostatic interactions and hydrogen bonding. These changes indicate interactions between the hydroxyl groups of alginate and functional groups from the bacteria (Obradović et al., 2015). Compared to corresponding composites, microparticle formulations (Samples 5, 6, and 7) show narrower and less intense bands in the region between 3000 and 3500 cm^−1^, indicating weakening of hydrogen bonding. The peaks at 2970 and 2943 cm^−1^ (assigned to -CH stretching in the asymmetric –CH_2_ stretching regions) are also less intense, indicating the influence of LB12 methyl and methylene groups specific to the bacterial cell wall fatty acids. Sample 5 shows lower peak intensities at 1609–1418 cm-1, indicating changes in the chemical environments of the carboxylate and amide groups. The peaks at 1032 cm^−1^ and 1170 cm^−1^ represent the symmetric and asymmetric stretching of the phosphoric acid in nucleic acids, together with the C-O-C deformation vibration from the polysaccharides (1200–900 cm^−1^) bonded to the glycopeptides and lipopolysaccharides of the cell wall (Han et al., 2020). Compared to their composites, samples 6 and 7 show narrower, less intense, and somewhat shifted characteristic bands, indicating reduced electrostatic interactions and hydrogen bonds. Visible changes are seen in the fingerprint region of LB12. In addition, in the polysaccharide region, lower peak intensities at 1315 and 1227 cm^−1^, along with specific vibrational features of bacterial proteins, are observed.

The FTIR spectra of microparticles and microparticle formulations reflect complex interactions among the functional groups of their constituents. The spectra are dominated by the main alginate bands, with crosslink formation via hydrogen bonding and electrostatic interactions, but not by covalent interactions. Higher intensity and wider absorption bands associated with stretching vibrations of O—H groups, as well as shifting of peaks associated with COO- groups and C-O-C groups, indicate an increase in hydrogen bonding and electrostatic interactions in Sample 4 compared to calcium alginate. However, encapsulating LB12 in composites weakens hydrogen bonds and electrostatic interactions, thereby altering the network structure.

#### 3.2.2 Swelling of microparticles

When calcium alginate comes into contact with an aqueous environment, it swells as the solution penetrates the matrix. Ionic crosslinking and hydrogen bonds have key, but distinct, roles in determining the swelling behavior of alginate hydrogels. The crosslinking of alginate with calcium ions creates a three-dimensional network structure, often described by the “egg-box” model, where mainly guluronic acid blocks in the alginate chains bind to calcium ions (Li et al., 2007). The number of ionic crosslinks formed between alginate chains and calcium ions per unit volume of the hydrogel determines the degree of polymer network connectivity. The crosslinking density mainly depends on the alginate composition and calcium ion concentration. Higher concentrations of calcium ions lead to a higher crosslinking density (Malektaj, 2023). Higher crosslinking density results in a more tightly packed alginate network, leading to reduced swelling. Alginate chains contain hydroxyl and carboxyl groups capable of forming hydrogen bonds. Hydrogen bonding influences swelling in alginate hydrogels through interactions with ionic crosslinking, controls water uptake, stabilizes the network structure, and responds to environmental conditions. Initially, adsorbed water is free water, not chemically bound to the alginate molecules. During swelling, some of the free water becomes bound water through hydrogen bonding and other interactions with the alginate molecules. Stronger hydrogen bonds with water promote swelling, whereas extensive interchain hydrogen bonds limit swelling by reducing the number of available water-binding sites (Wang et al., 2025).

Blending alginate with other polymers alters hydrogen bonding and electrostatic interactions, thereby modifying the hydrogel's properties (e.g., swelling, mechanical, thermal, and release behavior). Before the addition to the crosslinking solution, some interactions may occur in the mixture of components, between the functional groups of the two polysaccharides (hydroxyl, carboxylate, and sulfate) or polysaccharides and protein (carboxyl, amino, and phosphate), as well as with bacteria. These interactions include (i) hydrogen bonding and ionic interactions between polysaccharides, (ii) hydrogen bonding, ionic interactions, and hydrophobic interactions between protein and polysaccharides, and (iii) LB12 functional groups also contribute to the overall interactions among the components. In addition to alginate crosslinking, calcium ions can interact with other components, thereby altering the concentration of calcium available for crosslinking. These interactions include the binding of calcium ions to negatively charged sites on sodium caseinate molecules, the interaction of calcium ions with negatively charged sulfate groups on κ-carrageenan, and the binding of calcium ions to LB12 cells. The exact structure of an alginate-based composite and microparticle formulation depends on how all the composite components interact with each other.

The incorporation of CA, CAR, or CA and CAR into the calcium alginate gel matrix increased the degree of swelling (Table 1), indicating the increased distances between the crosslinked polymer chains (Postolović et al., 2022). This appears to be mainly attributed to the electrostatic repulsion between the negatively charged sites of alginate, casein, and κ-carrageenan and polymer-water hydrogen bonding. Reduced calcium ion availability to alginate chains also contributes to reduced crosslinking and, consequently, increased swelling. In addition to the degree of chain connectivity, swelling properties also depend on the spatial distribution of crosslinking sites in the composite gels (Shen et al., 2018). As shown in Table 1, the combination of components in the composites enhances swelling relative to calcium alginate. All microparticle formulations exhibit greater swelling than the corresponding composites, consistent with the FTIR data showing weakened hydrogen bonds and electrostatic interactions (Figs. 5a, b).

**Table 1:** Swelling (Sw) means ± SD (*n* = 100) with one-way ANOVA and Tukey HSD groupings (different letters = significantly different, α = 0.05).

Sample	S_w_ (mean ± SD, n = 100)	Group
Sample 0	53.3 ± 1.2	a
Sample 4	56.9 ± 1.1	b
Sample 2	76.5 ± 1.2	c
Sample 1	79.8 ± 1.1	d
Sample 5	80.5 ± 1.1	e
Sample 6	83.4 ± 1.2	f
Sample 3	114.6 ± 1.3	g
Sample 7	123.8 ± 1.3	h

ANOVA summary: one-way ANOVA, F(7, 792) ≈ 43,176, *p* < 0.0001; η2 ≈ 0.997.

Post-hoc (Tukey HSD): critical difference ≈ 0.611 (equal n = 100); all pairwise mean differences exceeded this threshold, so every pairwise comparison was statistically significant at α = 0.05 — group letters indicate distinct subsets.

A one-way ANOVA revealed a highly significant effect of formulation on swelling (S_w_) (F(7, 792) ≈ 43,176, *p* < 0.0001), with a very large effect size (η2 ≈ 0.997). Tukey HSD post hoc tests showed that all eight samples differed significantly from one another (all pairwise differences >0.611). Sample 7 (123.8 ± 1.3) exhibited the highest swelling, and Sample 0 (53.3 ± 1.2) the lowest; intermediate samples differed significantly from both extremes.3.2.3. Rheological properties of microparticles.

**Fig. 6:**
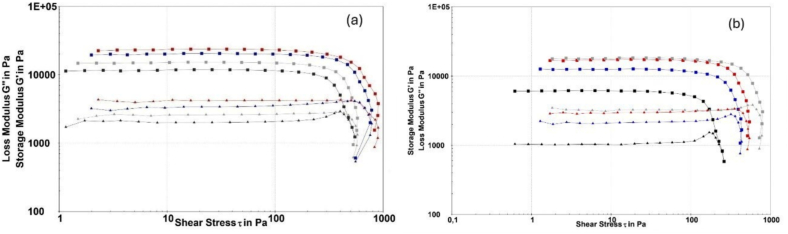
Amplitude sweep tests (G′ (■) and G″ (▲) values) of (a) Sample 0 (red), Sample 1 (gray), Sample 2 (blue) and Sample 3 (black) and (b) microparticle formulations (right side) and of Sample 4 (red), Sample 5 (gray), Sample 6 (blue) and Sample 7 (black) determined at a constant angular frequency of 10 rad/s at 23 °C. (For interpretation of the references to colour in this figure legend, the reader is referred to the web version of this article.)

The strength and stability during processing and application of alginate and alginate-based gels is closely related to their rheological properties. Their ability to exhibit both viscous and elastic properties, that is, viscoelasticity, makes them suitable for various applications. The viscoelastic properties were evaluated for all microparticle combinations of alginate, carrageenan, and casein, with and without LB12, using amplitude sweep tests; the results are presented in Figs. 6a and 6 b and in Table 2. The elastic portion of the material represents the storage modulus G′ (Pa), and the viscous portion represents the loss modulus (G"). The sample exhibiting the highest storage modulus was Sample 0, with a G′ value of 23,652 Pa. A slightly lower G′ value was observed for Sample 2. The corresponding yield stress values for these two systems were 404 Pa and 391 Pa, respectively.

**Table 2:** Results of Amplitude sweep tests of the calcium alginate (Sample 0), composites (Samples 1 to 3), and microparticle formulations (Samples 4 to 7) examined at 23 °C.

Microparticles	G′ (max)/Pa	SD	Yield point/	SD	Yield point/Strain/%	SD	Loss factor
			Stress/Pa				
Sample 0	23,652	1186	404.2	61.4	2.18	0.36	0.18⁎
Sample 1	15,134	1066	331.4	35.4	2.75	0.22	0.17
Sample 2	20,149	2287	391.1	67.3	2.38	0.18	0.16
Sample 3	11,822	1760	264.6	41.0	2.7	0.33	0.17
Sample 4	17,363	1115	269.3	32.6	1.95	0.17	0.17
Sample 5	18,259	967	384.4	34,7	2.72	0.18	0.18
Sample 6	12,523	1329	220.8	29,0	2.23	0.19	0.17
Sample 7	6134	678	127.6	15,0	2.65	0.28	0.17

⁎ The variability of the loss factor was negligible (CV ≈ 2%, SD typically ±0.002–0.004), therefore, standard deviations are not shown.

A notable trend is the progressive reduction in both storage modulus and yield stress with increasing composite complexity. Specifically, Sample 1 exhibited a G′ value of 15,134 Pa and a yield stress of 331 Pa, while the most complex composite, Sample 3, showed a further decrease in yield stress to 265 Pa. These results indicate that incorporating CA modifies the mechanical response of the polymer network. Despite these changes, the loss factor (tan δ) remained within a narrow range for all samples, suggesting a comparable balance between elastic and viscous contributions. For samples analyzed without bacterial cells, the storage modulus (G′) followed the order:

Sample 0 > Sample 2 > Sample 1 > Sample 3.

The correlation between storage modulus is consistent with the swelling degree presented in Table 1. Generally, higher crosslinking density leads to higher storage modulus and yield point, indicating a stiffer gel and greater resistance to deformation (Malektaj, 2023).

Statistical analysis using one-way ANOVA revealed significant differences among microparticle formulations for G′ max, yield stress, and yield strain (p<0.001). Tukey's HSD post hoc test demonstrated that Samples 0, 2, and 5 generally exhibited superior rheological performance, with significantly higher storage modulus and yield stress values compared with Sample 7. In contrast, Sample 4 showed significantly reduced yield strain, indicating lower deformability prior to structural failure.

With the addition of *Lactobacillus bulgaricus*, differences in viscoelastic behavior were observed across the microparticle formulations, indicating interactions between bacterial cells and the surrounding polymer matrix (Fig. 6b and Table 2). Compared with the corresponding cell-free samples, the presence of bacterial cells generally decreased both the storage modulus and the yield stress. An exception was observed for the CA formulation, in which slightly higher values were recorded for Sample 5. This behavior suggests that, in this specific system, bacterial cells preferentially interact with the protein component, contributing to partial structural stabilization. The viscoelastic properties of the microparticle formulations decreased in the following order:

Sample 5 > Sample 4 > Sample 6 > Sample 7.

Storage modulus values for these samples ranged from approximately 18,000 Pa to 6000 Pa, whereas the corresponding cell-free formulations ranged from 23,000 Pa to 12,000 Pa. This comparison indicates that incorporating bacterial cells alters the mechanical integrity of the composite networks. Although the swelling degree data (Table 1) indicated higher crosslinking density for Sample 4, rheological data for Sample 5 exhibited somewhat higher storage modulus and yield point. The mechanical behavior of alginate gels depends not only on the crosslinking density, but also on how the crosslinking is spatially distributed, as well as on hydrogen bonding. Two gels with the same overall crosslinking density can behave very differently if one has a uniform network and the other a non-uniform network structure (Shen et al., 2018). The spatial distribution of crosslinking sites in Sample 5 appears to be more uniform than in Sample 4, resulting in higher values of storage modulus and yield point. Shifted and more intense peak attributed to C=O stretching of carboxyl group around 1400–1450 cm^−1^ also suggests stronger interchain interaction in Sample 5 (Fig. 5b). The highest yield stress among the microparticle formulations was observed for Sample 5 (384.4 Pa), while Sample 7 exhibited the lowest yield stress (127.6 Pa). For comparison, the corresponding cell-free Sample 3 system displayed a yield stress of 264.6 Pa. Despite these differences, the loss factor values remained similar across all samples, indicating that the overall network organization is largely preserved.

##### 3.2.2.1 Frequency sweep

**Fig. 7:**
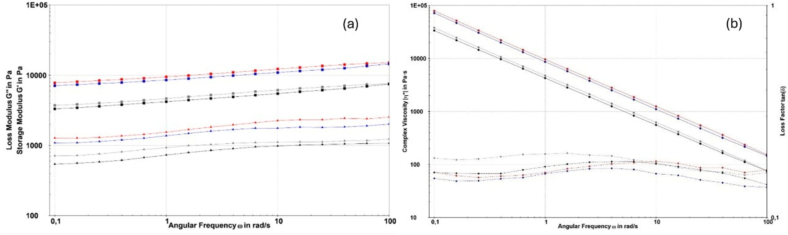
(a) Frequency sweep test (G′ (■) and G″ (▲) values) and (b) complex viscosity (ƞ*) and loss factor (tan δ) of Sample 0 (red), Sample 1 (gray), Sample 2 (blue) and Sample 3 (black) determined at a strain of 0.1% at 23 °C. (For interpretation of the references to colour in this figure legend, the reader is referred to the web version of this article.)

Frequency-dependent spectra determined for calcium alginate and composites are presented in Fig. 7. All samples exhibited stable viscoelastic behavior over a broad frequency range. They displayed shear-thinning behavior with comparable slopes, indicating similar flow properties across different microparticle compositions. The loss factor values varied within a narrow range from 0.1 to 0.2 across the applied frequency range, indicating a consistent degree of structural organization. Slightly higher loss factor values were observed at lower frequencies for Sample 1, which is consistent with trends observed in the amplitude sweep measurements. This behavior correlates with casein's influence on particle structure and morphology, resulting in a modest reduction in elastic response.

**Fig. 8:**
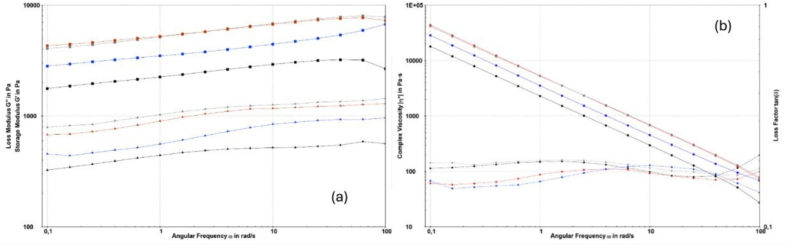
(a) Frequency sweep test (G′ (■) and G″ (▲) values) and (b) complex viscosity (ƞ*) and loss factor (tan δ) of Sample 4, Sample 5, Sample 6 and Sample 7 determined at a strain of 0.1% at 23 °C.

Frequency sweep measurements performed on samples containing LB12 revealed similar trends (Fig. 8). All microparticle formulations remained stable over a wide frequency range and exhibited shear-thinning behavior with comparable curve slopes. The loss factor again remained within a narrow range (0.1–0.2), with slightly elevated values at lower frequencies for casein-containing systems.

Overall, the most pronounced changes in viscoelastic behavior were associated with the incorporation of bacterial cells, followed by CA's influence on the complexity of interactions within the composite matrix. These effects are consistent with observations obtained from complementary characterization techniques.

#### 3.2.3 Thermal properties of microparticles

**Fig. 9:**
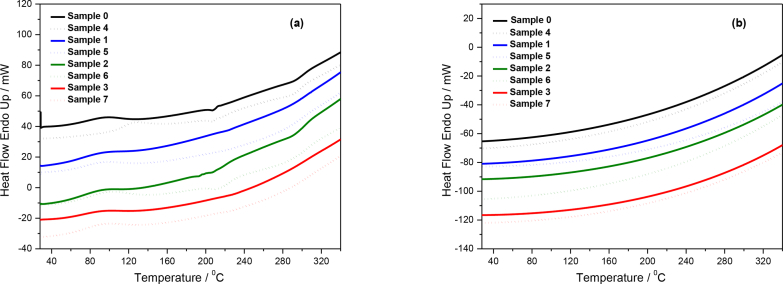
DSC curves of the first (a) heating cycle and (b) cooling cycle for microparticles. Samples are marked.

The thermal characteristics of microencapsulated probiotics are among the main factors affecting their stability during storage and application, and are also a key factor in bottom-up usability assessment. When probiotics are encapsulated in alginate gels or, even better, in their composites, their heat resistance during processing is significantly improved (Dong, Chen, Xin, Qin, Cheng, & Tang, 2013). Thermal stability testing of the prepared microparticles by differential scanning calorimetry showed three transitions in the heating curves (Fig. 9a) similar to the alginate composites from our previous studies (Vinceković et al., 2024). There were no thermal changes during the cooling cycles (Fig. 9b), indicating that the microparticles are mainly amorphous. All transition temperatures and enthalpies calculated from the first heating cycle at a heating rate of 10 °C/min are presented in Table 3.

**Table 3:** Transition temperatures, corresponding enthalpies (ΔH), and the change in heat capacity (∆Cp) of microparticles from the first heating cycle at a heating rate of 10 °C/min: Tendo (the first endothermic transition), Tg (the glass transition temperature), and T_cc_ (the cold crystallization temperature).

Microparticles	First transition	Second transition	Third transition
Sample 0	T_endo_ = 92.96 °C ΔH_endo_ = 100.08 J/g	T_g_ = 210.28 °C ΔCp = 3.03 J/gC	T_cc_ = 291.03 °C ΔH_cc_ = −72.68 J/g
Sample 1	T_endo_ = 80.35 °C ΔH_endo_ = 119.63 J/g	T_g_ = 214.15 °C ΔCp ∼ 1 J/gC	T_cc_ = 290.78 °C ΔH_cc_ = −37.98 J/g
Sample 2	T_endo_ = 91.75 °C ΔH_endo_ = 99.79 J/g	T_g_ = 220.45 °C ΔCp = 3.66 J/gC	T_cc_ = 292.2 °C ΔH_cc_ = −80.53 J/g
Sample 3	T_endo_ = 92.83°C ΔH_endo_ = 125.33 J/g	T_g_ = 220.54 °C -	T_cc_ = 292.5 °C ΔH_cc_ = −9.75 J/g
Sample 4	T_endo_ = 113.11 °C ΔH_endo_ = 109.87 J/g	Tg = 218.27 °C ΔCp = 3.17 J/gC	Tcc = 290.65 °C ΔH_cc_ = −83.82 J/g
Sample 5	T_endo_ = 92.06 °C ΔH_endo_ = 147.29 J/g	T_g_ = 214.56 °C ΔCp ∼ 1.13 J/gC	T_cc_ = 284.16 °C ΔH_cc_ = −36.66 J/g
Sample 6	T_endo_ = 95.21 °C ΔH_endo_ = 205.11.J/g	Tg = 213.55 °C ΔCp = 3.20 J/gC	T_cc_ = 291.94 °C ΔH_cc_ = −88.34 J/g
Sample 7	T_endo_ = 91.67 °C ΔH_endo_ = 187.59 J/g	T_g_ = 224.56 °C ΔCp = 1.3 J/gC	T_cc_ = 294.56 °C ΔH_cc_ = −18.62 J/g

The first broad endothermic peaks in the 70–120 °C range (for some samples, even broader) are associated with the release of free water during heating. Namely, alginate-based microparticles contain free water in macropores with increased mobility, as well as bound water molecules connected into a cross-linked gel network (El Nokab et al., 2024). The thermal stability of alginate-based gels at approximately 100 °C is an important factor in their application. The improved thermal stability allows probiotics to survive processing steps. The lowest peak temperature for the first transition of Sample 1, around 80 °C, can be attributed to the presence of CA, which typically has a glass transition temperature of around 100 °C (Kalichevsky et al., 1993). This value is higher than that for other commercial proteins used to prevent stickiness in foods, but in this case, the other components mask CA's glass transition temperature. Compared to the corresponding composites, all microparticle formulations exhibit higher temperature and enthalpy values for this broad endothermic transition, indicating that the presence of LB12 generally increases temperature stability and suggesting great potential for use. The highest enthalpy for the first transition was observed for Samples 6 and 7, indicating good thermal stability. This can be attributed to the hydrophilic properties of κ-carrageenan, which improve water absorption, as evidenced by the higher degree of swelling and consistent with the literature (Pascalău et al., 2012).

The second transition observed in the range of 210–225 °C corresponds to the glass transition temperature (T_g_) at which polymers transition from a glassy material to a rubbery state. This transition is a process that originates from the transition of the calcium alginate network into a gel and confirms the amorphous nature of all microparticles. Relatively high T_g_ values across all microparticles suggest that their stability was achieved before they undergo further structural changes upon heating (Gargallo & Radic, 2009). As shown in Table 3, the presence of LB12 increases the glass transition temperatures, except for Sample 6. Compared to the corresponding composite (Sample 2), the lower T_g_ value of Sample 6 may be attributed to the less restricted mobility of chain segments caused by the presence of bacteria. The highest T_g_ values observed in all samples with CA and CAR are attributed to electrostatic interactions between the –NH3+ of CA and the –OSO3- of CAR, as confirmed by FTIR analyses.

The exothermic peak in the third observed change is due to cold crystallization of calcium alginate gels before the final polymer degradation, including saccharide ring depolymerization. (Vinceković et al., 2024). In the thermal analysis of polymers, exothermic peaks typically occur between 284 and 294 °C, after the glass transition temperature (Gargallo & Radic, 2009). As microparticles undergo the glass transition, changes in the orientation of long or crosslinked polymer chains occur due to the material's softening, as seen in recrystallization, which can stabilize the polymer before it degrades. The enthalpy change of crystallization (ΔH_cc_) is the energy released during cold crystallization (the formation of crystalline regions when a polymer is heated after being previously annealed in an amorphous state) (Cai, 2013). In alginate-based composites, crystallization enthalpy change reflects how alginate affects the crystallization behavior of the polymer matrix (Malektaj, 2023). Compared to calcium alginate (Sample 0), the ΔH_cc_ values in samples containing carrageenan (Samples 2 and 6) were higher and lower in samples containing both casein and carrageenan (Samples 3 and 7). An increase in ΔH_cc_ in alginate-based composites typically indicates enhanced crystallinity (Cai, 2013). In almost all microparticle formulations, higher ΔH_cc_ values  than those observed in corresponding composites indicate a reduced phase transition energy due to LB12 encapsulation. The exceptions are samples with casein, which have approximately the same ΔH_cc_ values.

#### 3.2.4 Microscopic characterization of microparticles

Wet microparticles observed by light microscopy were approximately spherical in shape with some minor irregularities. Calcium alginate and composite microparticles were transparent, while those loaded with LB12 cells were white. The average size of calcium alginate microparticles was 780 ± 86 μm, and somewhat smaller compared to composites: Sample 1 (886 ± 76 μm), Sample 2 (901 ± 79), and Sample 3 (954 ± 61 μm). Microparticle formulations were somewhat larger than corresponding composites: Sample 4 (814 ± 86 μm), Sample 5 (914 ± 60 μm), Sample 6 (981 ± 90), Sample 7 (1001 ± 90 μm).

Particle size differed significantly among formulations (one-way ANOVA, F(7, 792) = 94.79, *p* < 0.0001, η2 = 0.456), indicating that formulation accounted for approximately 45.6% of the observed variance in particle size. Group means were: Sample 0 (calcium-alginate control) 780 ± 86 μm; Sample 1886 ± 76 μm; Sample 2901 ± 79 μm; Sample 3954 ± 61 μm; Sample 4814 ± 86 μm; Sample 5914 ± 60 μm; Sample 6981 ± 90 μm; Sample 71,001 ± 90 μm. Tukey HSD post-hoc comparisons (α = 0.05; HSD ≈ 28.8 μm) indicated that many pairwise differences were statistically significant. In particular, Samples 6 and 7 (981 and 1001 μm) and Sample 3 (954 μm) were significantly larger than the control (Sample 0, 780 μm) and most composites. Several adjacent means did not differ significantly: Sample 1 vs Sample 2 (difference 15 μm, ns), Sample 1 vs Sample 5 (28 μm, ns, just below the HSD), Sample 2 vs Sample 5 (13 μm, ns), Sample 3 vs Sample 6 (27 μm, ns), and Sample 6 vs Sample 7 (20 μm, ns). Overall, microparticle formulations tended to be larger than the corresponding composites, and Samples 6–7 showed the greatest increases in mean particle size. These results indicate that formulation composition substantially affects microparticle size, with several formulations producing significantly larger particles than the calcium-alginate control.

**Fig. 10:**
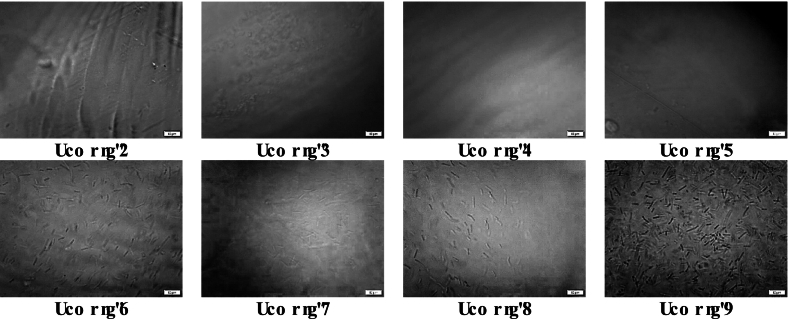
Representative OM microphotographs of wet microparticles. Samples and scales are marked.

At higher magnification, all microparticles exhibited relatively smooth surfaces with some subtle undulations or wrinkles (Fig. 10). Rod-like LB12 cells are located on the surface of microparticle formulations. Upon drying (on air at room temperature) to a constant mass, all microparticles decreased in size (by about 40%) and deformed due to polymer stress relaxation (Sayadi et al., 2024; Vinceković et al., 2024).

**Fig. 11:**
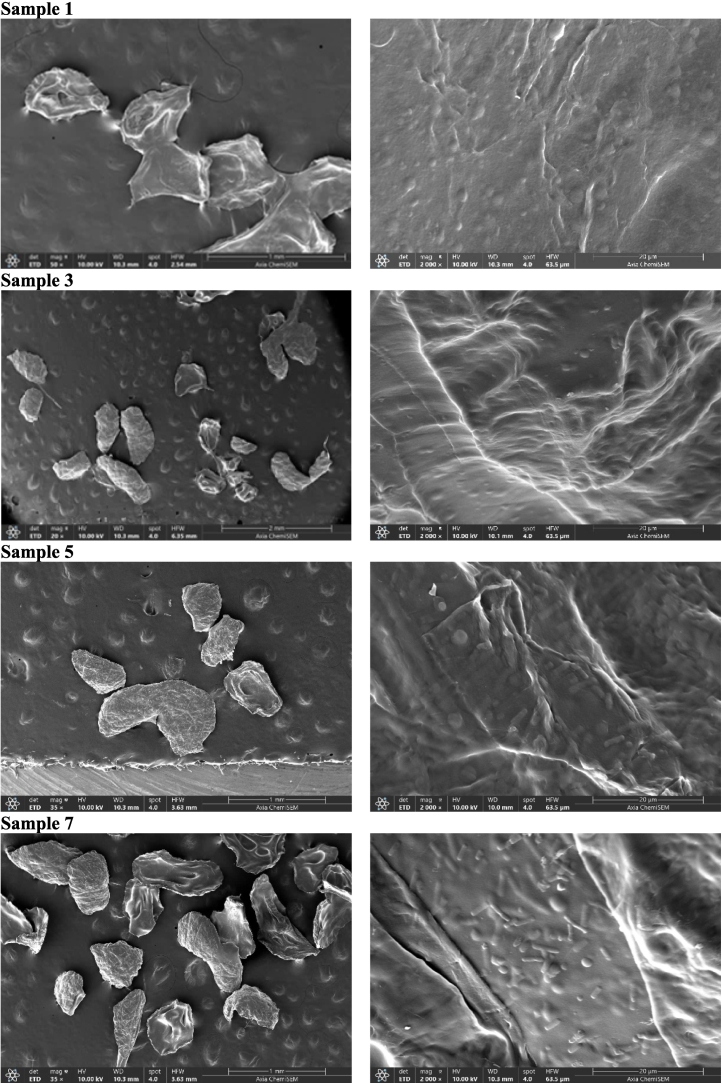
SEM microphotographs of lyophilized composites (Samples 1 and 3) and microparticle formulations (Samples 5 and 7) at different magnifications. The scale bars are indicated.

Based on the observed rheological and thermal data, Samples 5 and 7 were used for further experimentation. SEM micrographs revealed significant changes in the size and shape of microparticles after lyophilization (left column in Fig. 11). They became smaller and more deformed due to biopolymer stress relaxation as water was lost during lyophilization. Microparticles in Sample 1 showed the greatest deformation, revealing uneven, plate-like, and partially collapsed particles with broken sides. The microparticles in Sample 3 had a noticeable fibrous or reticular surface texture, indicating interpenetrating networks between polysaccharides and proteins (Li et al., 2021). Both microparticle formulations, Samples 5 and 7, showed fine protrusions or adhered particles, indicating the presence of embedded or surface-associated bacterial cells. The microparticles in Sample 7 appeared somewhat rougher with fewer detached fragments; surface irregularities were shallower and covered with a fibrous covering. Carrageenan can interact with alginate to create a porous and irregular surface, while casein can act as a surfactant, smoothing the surface (Khlibsuwan et al., 2018).

Under higher magnification, the microparticle surfaces exhibited pores and varied morphologies. The surface of Sample 1 appeared smooth with some cracks and an average pore size of 436 nm. Typically, a smooth surface appears when casein is mixed with alginate as the dominant phase, forming a bicontinuous phase, where both alginate and casein form interconnected networks (Li et al., 2021). Samples 1 and 3 differed significantly in morphology. The addition of carrageenan and casein to the alginate matrix likely plays a significant role in producing a heterogeneous, rougher surface with an average pore size of 628 nm. Each biopolymer contributes to the composite's final structure through interactions with the others. Alginate with carrageenan can form a composite interpenetrating double network (Postolović et al., 2022), casein and carrageenan can form gels with casein micelles distributed within carrageenan helices (Zhang et al., 2023), and casein networks can intertwine with alginate chains. Alginate/casein composite forms protein–polysaccharide networks with smoother surfaces, while alginate/casein/carrageenan composite exhibits rougher and more porous, polysaccharide-dominated structures.

When probiotic cells were included, Samples 5 and 7 showed a heterogeneous surface with rod-shaped LB12 cells present, which was similarly observed with the BB-12 cell strain (Vinceković et al., 2024). The surface of Sample 5 with ridges and valleys could be explained by the arrangement of polymer chains during crosslinking and by the presence of LB12 cells near the surface. The average pore size on the surface of Sample 5 was 716 nm, but the average pore size on the surface of Sample 876 nm. Sample 7 presents a more textured surface with distinct features. Rod-like bacterial structures were observed on the surface, but the presence of additional texture may indicate improved capture and stability of the LB12 cells (Vinceković et al., 2024). The functional groups of LB12 (hydroxyl, carboxyl, amino and phosphate groups) interact with the components of the composites, creating various complementary changes in the network structure and morphology of the calcium alginate. These findings show that both ionic crosslinking by Ca^2+^ and the presence of casein and carrageenan significantly affect particle microstructure, and that the addition of probiotic bacteria modifies surface topology—most likely through cell-matrix interactions. A better understanding of structure-property relationships can guide formulation selection, depending on whether protection or controlled release is the priority.

EDS spectra from representative particles and surfaces (the electron probe can penetrate to a depth of about 1 μm) confirmed the expected primary elements from the biopolymer constituents: C and O dominated all spectra (organic matrix), with detectable Ca signals. N was detected in composites and microparticle formulations containing casein, and S in those containing carrageenan. Minor Na and Cl peaks were observed in varying degrees and are most likely due to residual salts from the preparation or the buffer.

**Fig. 12:**
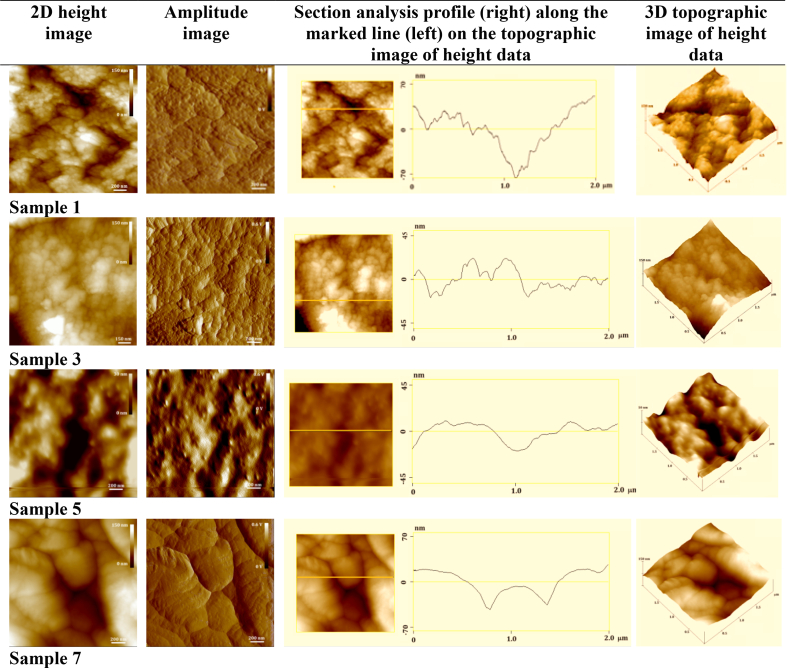
AFM microphotographs of composite microparticles (Sample 1 and Sample 3) and microparticle formulations (Sample 5 and Sample 7).

AFM analysis of 2D and 3D topographic height and phase images, along with surface cross-sectional profiles recorded along the lines shown in Fig. 12, provided deeper insight into the surface topology revealed by optical and SEM microscopy. The analysis revealed that the surface of Sample 1 is covered with grains ranging from 800 to 1000 nm in size. Between them are cavities up to 70 nm deep, which sharply separate individual grains from each other. The grains have a well-defined substructure, manifested as small, homogeneous, nearly spherical formations with a diameter of approximately 50 nm. By incorporating LB12 cells (Sample 5), the formulations' morphology is smoothed at the nanoscale, as evidenced by the loss of sharply separated grains with barely noticeable boundaries, and by indentations of only tens of nanometers.

The surface of Sample 3 exhibits a granular morphology with irregular grains, but the grain boundaries remain clearly recognizable. The surface profile clearly shows individual grains protruding from the microparticles' surfaces. Each grain has a substructural granular structure, but this is no longer as clearly defined as in microparticles without the addition of carrageenan to the formulation. The grains' surfaces are sharper and rougher than those of the microparticles without added carrageenan. The addition of LB12 cells (Sample 7) completely changes the surface morphology, and LB12 cells can be observed on the surface of the microparticles. The individual cells are clearly separated from each other by wells of up to 25 nm, indicating that they float on the surface of the microparticles.

#### 3.2.5 In vitro release of LB12 from microparticle formulations

Main processes involved in the release of encapsulated bioactive components from hydrophilic biopolymer microparticles are swelling, dissolution/erosion at the matrix surface, and diffusion through the matrix (Sriamornsak et al., 2007). The control mechanisms and release rates of hydrogel microparticles depend on the hydrogel composition, network microstructure, environmental conditions, and the properties of the encapsulated bioactive ingredient (Rahman et al., 2025). The crosslinking density of hydrogel microparticles significantly affects the release of encapsulated substances. Higher crosslinking density slows the release rate of encapsulated agents due to restricted movement through the microparticle matrix. Blending calcium alginate with other polymers alters the network structure, thereby affecting physical properties (mechanical, thermal, water uptake and permeability, stability, release properties, etc.). The exact structure of an alginate-based composite depends on how the ingredients interact with each other. The incorporation of LB12 into the alginate/casein gel matrix (Sample 5) alters the network structure and increases swelling relative to Sample 4. Factors that reduce crosslinking density are weakened hydrogen bonds between hydroxyl groups of alginate and amino acid residues and electrostatic repulsion between alginate chains and negatively charged casein, as well as chelation of calcium ions and the phosphate group of casein, which reduces the availability of free calcium for crosslinking in the alginate matrix (Vinceković et al., 2024). In the alginate/casein/carrageenan composite, the functional groups of each biopolymer contribute to ionic, hydrophobic, and hydrogen-bonding interactions, forming a complex multilayered network. The significantly higher S_w_ value  of Sample 7 can be attributed to the presence of a sulfate-containing polysaccharide, CAR, with pronounced hydrophilic properties (Mohamadnia et al., 2008). In addition, the binding of calcium ions to negatively charged sulfate groups on CAR chains reduces the availability of free calcium for crosslinking in the alginate matrix. The higher storage modulus and yield strength of Sample 5 suggest a stiffer gel and greater resistance to deformation than those of Sample 7. The microparticles of Sample 7 have higher values of encapsulation efficiency and loading capacity (EE = 41.0 ± 0.18%, LC = 8.2 × 10^11^ CFU g^−1^**)** compared to Sample 5 (EE = 35.0 ± 2.34%, LC = 4.5 × 10^11^ CFU g^−1^). Higher values of LC and EE for Sample 7 can be explained by differences in the structure of the microparticle network, which enables different accommodation of LB12 cells in the gel matrix, as well as by molecular interactions of LB12 functional groups (hydroxyl, amino, carboxyl, and phosphate) with calcium ions and functional groups of biopolymers in the microparticle formulations. The relatively lower encapsulation efficiencies observed in the present study can be explained by the complex interactions occurring within the multicomponent polymer systems. Unlike conventional calcium alginate matrices, the developed formulations simultaneously contained alginate, casein, κ-carrageenan, calcium ions, and LB12 cells, resulting in a highly interactive and structurally heterogeneous network. The incorporation of casein and κ-carrageenan introduced additional electrostatic interactions, hydrogen bonding, and competition for calcium ions, which influenced the classical calcium–alginate “egg-box” crosslinking mechanism. These interactions reduced the availability of free calcium ions for uniform alginate gelation and contributed to the formation of regions with different crosslinking densities throughout the microparticle structure. The formation of heterogeneous gel networks is further supported by swelling and rheological analyses, which demonstrated differences in viscoelastic behavior and water uptake among formulations. Increased swelling behavior indicates larger intermolecular spacing and less compact network organization, which can facilitate partial diffusion of bacterial cells from the matrix during microparticle formation and stabilization. Furthermore, the coexistence of alginate carboxyl groups, carrageenan sulfate groups, and casein phosphate and amino groups created a polyelectrolyte environment with non-uniform spatial distribution of intermolecular interactions, resulting in localized pores and structural heterogeneity within the gel matrix. The morphology of LB12 cells also likely contributed to the lower encapsulation efficiency. SEM and AFM analyses confirmed that LB12 exhibits a rod-shaped morphology with a tendency to form aggregates and short chains. Such elongated and clustered bacterial structures are more difficult to uniformly entrap within polymeric matrices compared to smaller or individually dispersed bacterial cells. During extrusion and ionic gelation, aggregated LB12 cells may partially localize near the microparticle surface, increasing the probability of bacterial leakage into the crosslinking medium before complete gel stabilization. In addition, the formulation strategy in this work focused primarily on achieving controlled-release behavior, swelling capacity, and compatibility with aqueous agricultural and hydroponic environments, rather than maximizing encapsulation efficiency alone. Lower polymer concentrations and milder gelation conditions were intentionally employed to preserve bacterial functionality and enable the gradual release of LB12 during application in water-based cultivation systems. Therefore, the encapsulation efficiencies obtained reflect a compromise among bacterial retention, hydrogel swelling behavior, structural flexibility, and the controlled-release performance required for the intended green biofertilizer application.

**Fig. 13:**
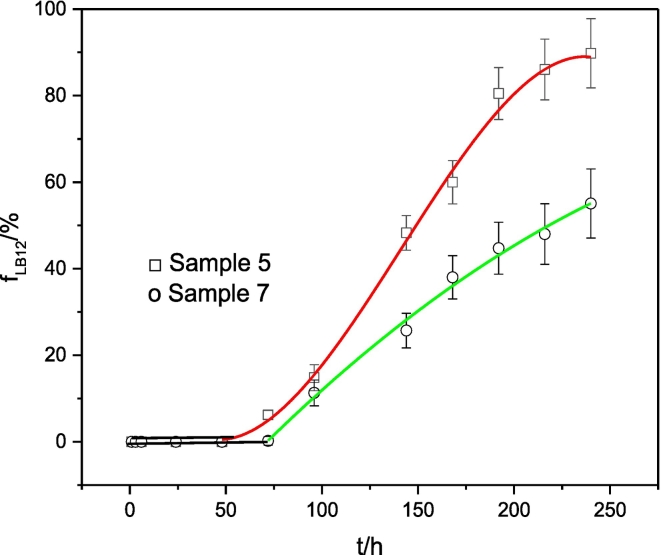
Fraction of cumulatively released LB12 cells from microparticle formulations (f_LB12_) with time (t). Error bars indicate the standard deviation of the means. Samples are denoted.

The release profiles of LB12 cells from samples 5 and 7 dispersed in distilled water show that after an initial lag time, the release follows a power law equation (Fig. 13). The lag phase is the time required for microparticles to hydrate, as the surrounding solution penetrates the pores on the surface, and for active substances to be transported through the microparticle matrix without being released into the surrounding medium. The lag phase of bacterial cells released from biopolymer microparticles can be influenced by several factors. These include the type of biopolymer, the release mechanism, the initial cell concentration, and the specific Lactobacillus strain (Rezvani et al., 2017). The obtained values of released LB12 cells should primarily be interpreted as comparative indicators of release behavior between formulations rather than as absolute viable cell counts.

The release of LB12 was analyzed by a modified empirical Korsmeyer-Peppas model (Kim & Fassihi, 1997) including the delay phase (Korsmeyer et al., 1983) as shown in Eq. (1):

(1) $$f_{\mathrm{LB}12}=k{\left(t-t_{l}\right)}^{n},$$

where f_LB12_ is the fraction of cumulatively released LB12, *k* is a kinetic constant characteristic for a particular system considering structural and geometrical aspects, *n* is the release exponent representing the release mechanism, t is the release time, and t_l_ is the lag time.

The release rate of active substances from hydrophilic biopolymers can be governed by three different mechanisms; (i) Fickian diffusion (diagnosed with *n* < 0.45), (ii) the anomalous transport kinetics (a combination of the two diffusion mechanisms and Type II transport (diagnosed with 0.45 > *n* < 0.89) and (iii) Type II transport, involving swelling and relaxation of the polymeric matrix (diagnosed with *n* > 0.89) (Azadi et al., 2017; Costa & Lobo, 2001). The values of the release constants *k* and exponents *n* are listed in Table 4. The *n* value for Sample 5 indicates that the LB12 cells' release-controlling process is diffusion through microparticles, whereas for Sample 7, it is swelling and relaxation of the polymeric matrix. A faster release rate and a higher number of LB12 cells released were observed in Sample 5, despite its higher degree of swelling, larger LC, and larger pore size compared with Sample 7. This may be attributed to differences in the mechanisms controlling LB12 release. The release of a larger amount of LB12 over a shorter time gives Sample 5 an advantage for various fermentation processes, while the slower release of LB12 from Sample 7 is suitable for the slow release biofertilizer in agricultural application. A slower release is desirable for probiotic survival in the soil and water environment and for safe delivery to the rooth of the plants.

**Table 4:** Release parameters of LB12 from microparticles in distilled water at 25 °C. *k* is a kinetic constant, *n* is the release exponent representing the release mechanism, and R^2^ is a correlation coefficient.

Microparticle formulations	*k/*h	*n*	R^2^
Sample 5	12.1 ± 0,65	0.43 ± 0,01	0.99
Sample 7	0.70 ± 0,15	0.96 ± 0,05	0.99

Encapsulation efficiency (EE), loading capacity (LC), and release-kinetic parameters (k/h and n) differed significantly between Sample 5 and Sample 7 (*n* = 100 per group). Sample 7 exhibited higher EE and LC than Sample 5 (EE: 41.0 ± 0.18% vs 35.0 ± 2.34%; one-way ANOVA F(1,198) = 653.5, *p* < 0.0001, η2 = 0.767; Tukey HSD p < 0.0001). LC was also greater for Sample 7 (8.2 × 10^11 ± 2.5 × 10^11 CFU g-1) than for Sample 5 (4.5 × 10^11 ± 1.2 × 10^11 CFU g-1; F(1,198) = 178.2, p < 0.0001, η2 = 0.474; Tukey HSD p < 0.0001). Release-kinetic parameters differed markedly between formulations. The k/h value for Sample 5 (12.10 ± 0.65) was substantially higher than for Sample 7 (0.70 ± 0.15; F(1,198) ≈ 2.92 × 10^4, p < 0.0001, η2 = 0.993; Tukey HSD p < 0.0001). The diffusional exponent n was lower for Sample 5 (0.43 ± 0.01) than for Sample 7 (0.96 ± 0.05; F(1,198) = 10,799, p < 0.0001, η2 = 0.982; Tukey HSD p < 0.0001). Based on the statistical analysis presented, Sample 7 showed significantly improved encapsulation (EE, LC) and markedly different release-kinetic behavior (lower k/h and higher n) compared with Sample 5, with large effect sizes across all comparisons.

## 4 Concluding remarks

A key property of the application of biopolymer microparticles, especially those composed of alginate-based composites, is directly related to controlled release under certain physicochemical conditions. Achieving prolonged or targeted release of encapsulated bacteria often requires simultaneous adjustment of the physicochemical properties of selected biopolymers. By adjusting the network structure, it is possible to achieve a more controlled and prolonged release of encapsulated bacteria, which is useful for applications in agricultural plnat cultivation and functional food productions. Alginate-based composites, alginate/casein, alginate/κ-carrageenan and alginate/casein/carrageenan, and corresponding formulations with encapsulated *Lactobacillus bulgaricus* cells were prepared by ionic gelation. Complex molecular interactions between alginate, casein, carrageenan and bacterial cells, before and during cross-linking, determine the structure of the alginate-based network. The broadening, shifting, or merging of FTIR peaks indicates the formation of a crosslinked alginate composite system mainly through hydrogen bonding and electrostatic interactions. However, rheological measurements indicate that the formation of multiple interaction sites does not necessarily lead to increased macroscopic elastic strength. Instead, the coexistence of several polymer-polymer and polymer-cell interactions appears to promote a network rearrangement that favors structural flexibility over stiffness, as reflected in reduced storage modulus values in more complex systems.

The rheological properties of alginate-based microparticles provide a macroscopic validation of the structural and physicochemical features identified by thermal analyses, microscopy, spectroscopy, and release studies. Variations in storage modulus and yield stress across formulations are consistent with morphological differences observed by optical microscopy, SEM, and AFM, in which increasing formulation complexity and bacterial incorporation led to more heterogeneous, less compact microparticle surfaces. Samples exhibiting smoother, denser surface layers showed higher yield stresses, indicating a more cohesive, mechanically stable network. In contrast, formulations characterized by increased surface irregularities and porosity exhibited lower yield stresses, consistent with a more compliant viscoelastic response. Such microstructural heterogeneity reduces the efficiency of stress transfer within the polymer network, thereby diminishing mechanical resistance under oscillatory deformation.

However, rheological measurements indicate that creating multiple interaction sites does not necessarily lead to increased elastic strength at the macroscopic level. Instead, the coexistence of several polymer–polymer and polymer–bacterial cell interactions appears to promote network rearrangement in favour of structural flexibility rather than stiffness, which is reflected in the reduced storage modulus values for the microparticle formulations.

All composites and microparticle formulations remained stable up to 80–100 °C, indicating strong potential for use. The presence of LB12 generally increased the temperature and enthalpy change of the first endothermic transitions, allowing probiotics to survive processing steps that would otherwise kill them.

The rheological curves are further supported by the release behavior of encapsulated *Lactobacillus bulgaricus*. The alginate/casein/carrageenan/LB12 formulation, which exhibits lower storage modulus and yield strength, corresponds to network structures that enable prolonged, controlled cell release. The alginate/casein/LB12 samples retained higher viscoelastic parameters upon bacterial incorporation, consistent with their smoother morphology and lower encapsulation efficiency. The release profiles of the probiotic bacteria indicated that Fickian diffusion is the rate-controlling mechanism for alginate/casein microparticle formulations. A rate-controlling mechanism for the release of bacterial cells from microparticle formulations with alginate/casein/κ-carrageenan involves swelling and relaxation of the polymeric matrix. Differences in the mechanisms controlling probiotic release indicate preferential use of the formulation in application. The release of a larger amount of LB12 in a shorter time gives the alginate/casein formulation an advantage for use in various fermentation and biofertilizer applications, while the slower release of LB12 from the alginate/casein/κ-carrageenan formulation is more desirable for prolonged and controlled release of bacterial cells in aqueous environments during agricultural and hydroponic applications.Overall, the combined analysis shows that, although local interactions and microstructural features govern particle morphology and molecular organization, macroscopic viscoelastic behavior is primarily determined by network continuity and load distribution. The progressive decrease in mechanical strength with increasing compositional complexity highlights the balance between structural stability and functional performance, which is crucial for designing biopolymer scaffolds with tailored mechanical and release properties for the application in agricultural plant cultivation.

## CRediT authorship contribution statement

**Marko Vinceković:** Writing – review & editing, Writing – original draft. **Botagoz Mutaliyeva:** Resources, Project administration. **Sanja Kajić:** Investigation, Formal analysis. **Nataša Šijaković Vujičić:** Formal analysis, Data curation. **Anđela Pustak:** Investigation, Formal analysis. **Suzana Šegota:** Methodology, Investigation, Formal analysis. **Tanja Jurkin:** Formal analysis, Data curation. **Lana Živković-Gemzić:** Investigation, Formal analysis. **Assem Issayeva:** Methodology, Investigation, Formal analysis. **Galiya Madybekova:** Resources, Project administration, Methodology, Investigation. **Usen Akhanov:** Investigation, Formal analysis.

## Informed consent statement

Not applicable.

## Institutional review board statement

Not applicable.

## Funding

This research is funded by the Science Committee of the Ministry of Science and Higher Education of the Republic of Kazakhstan (Grant No. AP23488050).

## Declaration of competing interest

The authors declare that they have no known competing financial interests or personal relationships that could have appeared to influence the work reported in this paper.

## Data Availability

Data will be made available on request.
